# A homeostatic function of CXCR2 signalling in articular cartilage

**DOI:** 10.1136/annrheumdis-2014-205546

**Published:** 2014-08-18

**Authors:** Joanna Sherwood, Jessica Bertrand, Giovanna Nalesso, Blandine Poulet, Andrew Pitsillides, Laura Brandolini, Alexandra Karystinou, Cosimo De Bari, Frank P Luyten, Costantino Pitzalis, Thomas Pap, Francesco Dell'Accio

**Affiliations:** 1 Centre for Experimental Medicine and Rheumatology, William Harvey Research Institute, Barts and the London School of Medicine and Dentistry, Queen Mary University of London, London, UK; 2 Institute of Experimental Musculoskeletal Medicine, University Hospital Muenster, Muenster, Germany; 3 Division of Medicine, Centre for Rheumatology and Connective Tissue Disease, UCL, London, UK; 4 Department of Veterinary Basic Sciences, Royal Veterinary College, University of London, Royal College Street, London, UK; 5 Dompé S.P.A., L'Aquila, Italy; 6 Institute of Medical Sciences, University of Aberdeen, UK; 7 Skeletal Biology and Engineering Research Center, KU Leuven, Belgium

**Keywords:** Chondrocytes, Chemokines, Osteoarthritis

## Abstract

**Objective:**

ELR+ CXC chemokines are heparin-binding cytokines signalling through the CXCR1 and CXCR2 receptors. ELR+ CXC chemokines have been associated with inflammatory arthritis due to their capacity to attract inflammatory cells. Here, we describe an unsuspected physiological function of these molecules in articular cartilage homeostasis.

**Methods:**

Chemokine receptors and ligands were detected by immunohistochemistry, western blotting and RT-PCR. Osteoarthritis was induced in wild-type and CXCR2^−/−^ mice by destabilisation of the medial meniscus (DMM). CXCR1/2 signalling was inhibited *in vitro* using blocking antibodies or siRNA. Chondrocyte phenotype was analysed using Alcian blue staining, RT-PCR and western blotting. AKT phosphorylation and SOX9 expression were upregulated using constitutively active AKT or SOX9 plasmids. Apoptosis was detected by terminal deoxynucleotidyl transferase dUTP nick end labelling (TUNEL) assay.

**Results:**

CXCL6 was expressed in healthy cartilage and was retained through binding to heparan sulfate proteoglycans. CXCR2^−/−^ mice developed more severe osteoarthritis than wild types following DMM, with increased chondrocyte apoptosis. Disruption of CXCR1/2 in human and CXCR2 signalling in mouse chondrocytes led to a decrease in extracellular matrix production, reduced expression of chondrocyte differentiation markers and increased chondrocyte apoptosis. CXCR2-dependent chondrocyte homeostasis was mediated by AKT signalling since forced expression of constitutively active AKT rescued the expression of phenotypic markers and the apoptosis induced by CXCR2 blockade.

**Conclusions:**

Our study demonstrates an important physiological role for CXCR1/2 signalling in maintaining cartilage homeostasis and suggests that the loss of ELR+ CXC chemokines during cartilage breakdown in osteoarthritis contributes to the characteristic loss of chondrocyte phenotypic stability.

## Introduction

Osteoarthritis (OA) is a leading cause of chronic disability, characterised by the breakdown of the articular cartilage, for which we have no cure. Whereas in inflammatory arthritides inflammation is the main driver of tissue destruction, in OA, mechanical factors are the main drivers of cartilage breakdown while a low degree of synovitis detected only in a subset of patients may have an ancillary role.^1^

Metalloproteinase and aggrecanase-mediated extracellular matrix (ECM) degradation, and chondrocyte apoptosis all contribute to cartilage breakdown and are initially compensated by chondrocyte proliferation and upregulation of SOX9, which directly regulates the synthesis of major ECM components including aggrecan and type II collagen.^2^^–^4 When such compensatory mechanisms are impaired or insufficient, cartilage breakdown progresses and ultimately leads to joint failure. Supporting the homeostatic response of cartilage can slow down or even revert cartilage degeneration in animal models.^5^ ^6^

Enclosed within the cartilage matrix, chondrocytes are not known to migrate in physiological conditions. In spite of this, however, chondrocytes express several chemokine receptors, including CXCR1 and CXCR2 and their cognate ligands that have been extensively studied in the context of arthritis.^7^ ^8^

ELR+ CXC chemokines are chemotactic cytokines characterised by their glutamic acid-leucine-arginine (ELR+) motif. The chemokine receptor CXCR2 binds the human CXC chemokine ligands CXCL1, CXCL2, CXCL3, CXCL5, CXCL6, CXCL7 and CXCL8 while CXCR1 binds only to CXCL6 and CXCL8.^9^ Mice lack *Cxcl8* and express only one gene that shows homology to the human CXCL5 and CXCL6 (hereafter referred to as mouse CXCL6). Although a putative murine homologue of human CXCR1 has been identified,^10^ mouse CXCR2 is considered the main ELR+ CXC chemokine receptor because its function cannot be compensated in neutrophil chemotaxis and wound healing.^11^^–^13 CXCR1 and CXCR2 activate intracellular calcium and lead to activation of the Pi3K/AKT signalling pathway.^14^ ^15^

Although the biology and expression *in vivo* of CXCR1 and CXCR2 in intact normal human articular cartilage has not been reported, the expression of these receptors and various chemokines have been characterised in isolated arthritic chondrocytes.^7^ ^8^ ELR+ CXC chemokines have been studied and targeted in inflammatory arthritis because of their capacity to attract inflammatory cells. CXCL8 was shown to stimulate, *in vitro*, the production of inflammatory mediators, metalloproteinases and the induction of hypertrophy and calcification.^16^

Here, we report a novel, unsuspected homeostatic role for CXCR1/2 signalling in the articular cartilage where ELR+ CXC chemokines are retained in the ECM through binding to GAGs and cell-autonomously support chondrocyte viability and differentiation through AKT-dependent SOX9 expression. We suggest that disruption of CXCR1/2 signalling is an important event in osteoarthritis, resulting in the loss of chondrocyte phenotypic stability and promoting OA-like changes.

## Materials and methods

### Mice

000.651 BALB/cJ and C.129S2(B6)-Cxcr2tm1Mwm/J CXCR2(−/−) mice (BALB/cJ background) were obtained from The Jackson Laboratory and maintained in pathogen-free conditions. The Animal Use Committee for the University of Münster (Landesamt für Natur, Umwelt und Verbraucherschutz, approval number 84-02.04.2012.A189) approved all mouse procedures.

### Destabilisation of the medial meniscus

Ten-week-old BALB/C or CXCR2^−/−^ male mice received destabilisation of the medial meniscus (DMM) and sham surgery to the contralateral limb as described.^17^

After 8 weeks, mice were killed and the joints were processed as previously described.^18^ A minimum of five sections per joint were stained using toluidine blue for histological analysis and Osteoarthritis Research Society International (OARSI) scoring for osteoarthritis severity by two independent investigators.

### Cartilage harvest, chondrocyte isolation and culture

Full-thickness human articular cartilage was obtained from the femoral condyles of patients undergoing knee joint replacement surgery (ethics approval from the East London & The City Ethics Committee 3). Normal articular cartilage was obtained from postmortem and trauma surgery donors. Chondrocytes were isolated and cultured as previously described.^19^ Mouse costal chondrocytes were obtained from the ribcages of BALB/C wild-type and CXCR2^−/−^ mice.

Micromass culture of primary chondrocytes or JJ012 cells and quantification of glycosaminoglycan content were performed as described.^20^ The ATDC5 cell line was differentiated in monolayer culture for 14 days in Dulbecco's modified Eagle's medium (DMEM) supplemented with 10 µg/mL human insulin as described^21^ before use.

### CXCR blockade using blocking antibodies

Chondrocyte culture medium was replaced with DMEM supplemented with 1% heat-inactivated fetal bovine serum. After 24 h, CXCR1 and CXCR2 blocking antibodies (R&D systems) or isotype-matched negative control antibodies (Dako) were added at a total concentration of 10 μg/mL. Chondrocytes were then cultured for 4 days before phenotypic analysis.

### siRNA transfection

Human CXCR1 and CXCR2, or mouse CXCR2, were knocked down using Stealth siRNA (Life Technologies), used at a total concentration of 20 nM in complete DMEM using jetPRIME transfection reagent (Polyplus) according to the manufacturer's instructions. A Stealth RNAi negative control duplex of medium guanine–cytosine (GC) content (Life Technologies) was used as a negative control.

### Western blot analysis

Cell lysates were run on a 10% Tris-glycine gel (Life Technologies) and transferred onto nitrocellulose membrane (GE Healthcare). Primary antibodies used were rabbit anti-mouse pAKT (ser473) (Cell Signaling) 1:200 dilution, rabbit anti-mouse AKT (Cell Signaling) 1:500 dilution or rabbit anti-mouse CXCL6 (Biorbyt) 1:200 dilution in blocking solution at 4°C overnight. For more detailed protocols, see online supplementary methods.

### Immunofluorescence analysis of mouse and human cartilage

Human cartilage explant and decalcified mouse knee joint sections were deparaffinised and the subsequent steps including blocking and pepsin antigen retrieval were performed as previously described.^22^ Human sections and monolayer cells were analysed using (anti-CXCR1, -CXCR2 or CXCL6 (R&D)) primary antibodies followed by Cy2 conjugated goat anti-mouse IgG secondary antibodies (Jackson ImmunoResearch). Mouse knee sections were incubated with rabbit anti-mouse -GCP2 (Biorbyt), -CXCR2 (R&D), -Col X (Abcam) or anti-Col II (Chemicon), followed by chicken anti-rabbit Alexa Fluor 488 or chicken anti-rabbit Alexa Fluor 594 secondary antibodies (Life Technologies). Mouse isotype control IgG (Dako) or rabbit IgG (Abcam) were used as control primary antibodies.

Apoptosis was detected by terminal deoxynucleotidyl transferase dUTP nick end labelling (TUNEL) assay (Roche) according to the manufacturer's instruction.

Images were acquired at 22°C by either Leica DM5500 Q Confocal microscope using 40× magnification/0.75 numerical aperture, or Olympus BX61 microscope with a fixed exposure using either 10×/0.4 or 20×/0.7 objective lenses using Cell-P software. Images were enhanced using Adobe Photoshop for better rendering without altering relationship of target to control images.

### Cartilage explant digestion

Hip caps from 4-week-old BALB/C wild-type mice were decellularised by repeated freeze thawing. Hip caps were incubated overnight at 37°C either in phosphate buffered saline (PBS) or in PBS containing 5 mU/mL heparitinase (Seikagaku). Total proteins were precipitated from supernatants using trichloroacetic acid and assessed by western blotting.

### Total RNA extraction and real-time RT-PCR

RNA extraction and gene expression analysis were performed as previously described,^19^ with additional primers found in online supplementary table S1.

### caAKT and SOX9 plasmid transfection

Mouse primary chondrocytes and ATDC5 cells were transfected in monolayer using jetPRIME (Polyplus) according to the manufacturer's instructions. caAKT (Addgene plasmid 10841)^23^ was used to constitutively activate AKT signalling, and a SOX9 plasmid was used to overexpress SOX9.^24^

### Statistical analysis

Data are presented as means±SEM. According to data distribution, parametric (Student t test) or non-parametric (Mann–Whitney) tests were performed using GraphPad Prism Software, V.5.0c (GraphPad Software Inc, San Diego, USA), with p<0.05 determining the primary level of significance.

## Results

### CXCR1/2 and their ligand CXCL6 are expressed in adult articular cartilage

**Figure 1:**
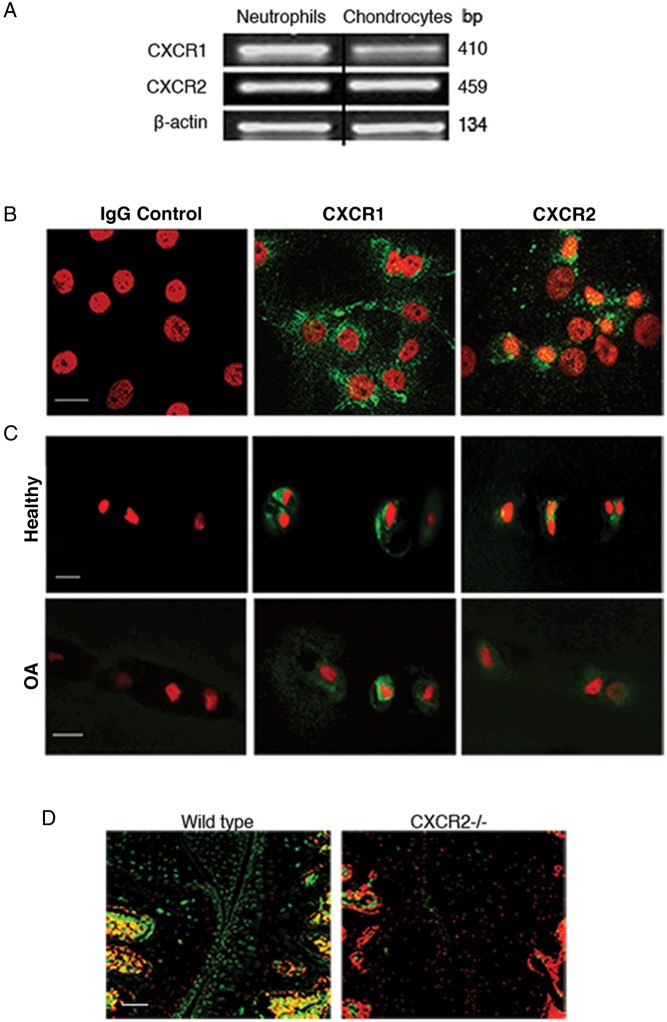
ELR+ CXC chemokine receptors are expressed in human and mouse articular cartilage. (A) Semi-quantitative RT-PCR for CXCR1 and CXCR2 in human primary chondrocytes cultured in monolayer. (B) Confocal microscopic image of immunofluorescence staining for CXCR1 and CXCR2 (green) in human primary articular chondrocytes cultured in monolayer, with propidium iodide (red) staining the nuclei. Scale bar, 20 μm. (C) Immunofluorescence staining for CXCR1 and CXCR2 (green) in normal and osteoarthritic articular cartilage, with propidium iodide (red) staining the nuclei. Scale bar, 20 μm. (D) Immunofluorescence staining for CXCR2 (green) in wild type and CXCR2^−/−^ mouse articular cartilage, with propidium iodide (red) staining the nuclei. Scale bar, 100 μm.

*In vitro*, primary adult human articular chondrocytes (AHAC) expressed the CXC chemokine receptors CXCR1 and CXCR2 at mRNA (figure 1A) and protein levels (figure 1B). We confirmed the expression of CXCR1 and CXCR2 within native human articular cartilage using immunohistochemistry showing that CXCR1 and CXCR2 were expressed in normal and osteoarthritic cartilage (figure 1C). CXCR2, the main functional murine ELR+ CXC chemokine receptor,^12^ ^13^ was expressed in normal mouse articular cartilage (figure 1D).

**Figure 2:**
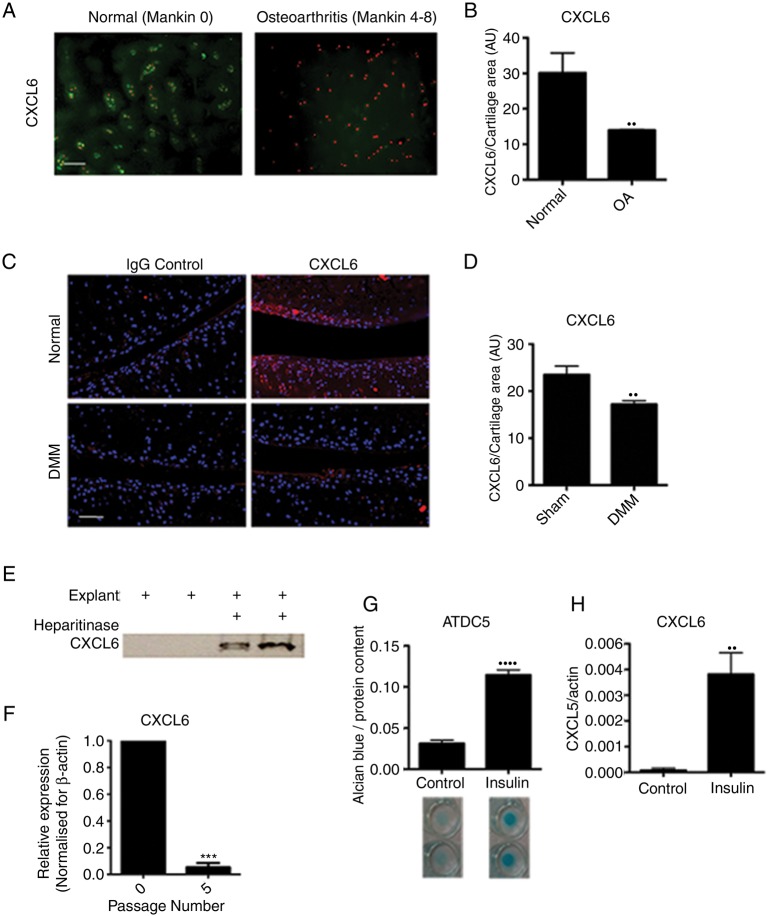
CXCL6 is present in healthy articular cartilage and its expression is associated with chondrocyte differentiation. (A) Immunofluorescence staining for CXCL6 (green) in normal and early osteoarthritis (moderate Mankin score) articular cartilage. Nuclei are stained using propidium iodide (red). Scale bar, 100 μm. (B) Densitometric quantification of CXCL6 staining (n=3). (C) Immunofluorescence staining for CXCL6 (red) in mouse articular cartilage of sham-operated control and destabilisation of the medial meniscus (DMM) operated mice, with 4′,6-diamidino-2-phenylindole staining the nuclei. Scale bar, 100 μm. (D) Densitometric quantification of CXCL6 staining (n=4). (E) Western blot analysis of CXCL6 release into supernatant from vehicle control or heparitinase treated, freeze-thawed wild-type mouse hip caps. (F) Real-time RT-PCR for CXCL6 mRNA in early and late passage human articular chondrocytes (n=3), ***p<0.001 by paired t test. (G) Alcian blue staining and spectrophotometric quantification of ATDC5 cell micromasses differentiated for 14 days using insulin (n=6). (H) Real-time RT-PCR quantification of CXCL6 mRNA expression in ATDC5 cells following 14 days of culture in either control or insulin supplemented differentiation medium (n=6) **p<0.01, ****p<0.0001.

The CXCR1/2 ligand CXCL6 had a striking and specific expression pattern in normal cartilage tissue, conserved across the mouse and human species (figure 2A, B). In humans, CXCL6 was found within the chondrocyte territorial matrix of articular cartilage from healthy donors; however, it could no longer be detected in the matrix of early osteoarthritic cartilage (figure 2A, B). This pattern was confirmed in the mouse, where CXCL6 was present within the articular cartilage matrix of unchallenged BALB/C mice, but was reduced 8 weeks following DMM surgery (figure 2C, D). In contrast, CXCL8 in human and CXCL1 in mouse were hardly detectable at protein level in healthy or OA cartilage (see online supplementary figure S1).

If high levels of CXCL6 are produced by healthy chondrocytes, what mechanism prevents them from attracting inflammatory cells in physiological conditions? ELR+ CXC chemokines are known to bind to heparan sulfate proteoglycans (HSPGs),^25^^–^27 therefore we hypothesised that binding to HSPGs within the avascular cartilage could retain them within the ECM and avoid their availability to inflammatory cells. To establish whether CXCL6 is retained within the cartilage ECM through binding to HSPGs, mouse cartilage explants were decellularised through freeze-thawing and were incubated overnight in the presence or absence of heparitinase. In keeping with its postulated binding to HSPGs, CXCL6 was subsequently retrieved from the supernatants of heparitinase digested explants but not from control undigested explants (figure 2E).

*In vitro*, in addition to CXCR1 and CXCR2 (figure 1), AHAC expressed CXCL6 (figure 2F) and CXCL8 (online supplementary figure 1D) mRNA at early passage, but not following serial passaging. Serial passaging is known to be associated with the loss of chondrocyte phenotypic markers—a process known as ‘dedifferentiation’^28^^–^30—and of their capacity to form cartilage *in vivo*.^28^ Conversely, chondrogenic differentiation of the mouse chondrocytic cell line ATDC5 using insulin (figure 2G) resulted in the upregulation of CXCL6 (figure 2H). Taken together, these data suggested that ELR+ CXC chemokine signalling may have a physiological function in chondrocyte homeostasis.

### CXCR2 deficiency results in more severe osteoarthritis following surgically induced joint instability

**Figure 3:**
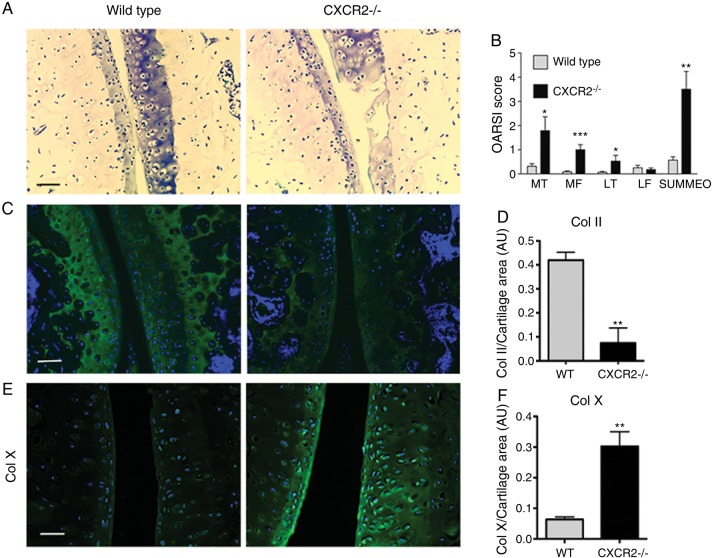
CXCR2 is required for articular cartilage homeostasis. (A) Toluidine blue staining for wild-type and CXCR2^−/−^ mouse articular cartilage 8 weeks following destabilisation of the medial meniscus (DMM) surgery. Scale bar, 100 μm. (B) Osteoarthritis Research Society International score of osteoarthritis-like changes and cartilage breakdown in BALB/C wild-type and CXCR2^−/−^ mice 8 weeks following DMM surgery (n=10). MT, medial tibial plateau; MF, medial femoral head, LT, lateral tibial plateau; LF, lateral femoral head. Statistical comparison using Mann–Whitney U test. (C) Type II collagen immunofluorescence staining of wild-type and CXCR2−/− mouse articular cartilage 8 weeks following DMM. Nuclei are counterstained with 4′,6-diamidino-2-phenylindole (DAPI). Scale bar, 100 μm. (D) Densitometric quantification of type II collagen staining in articular cartilage following DMM (n=4). (E) Type X collagen immunofluorescence staining of wild-type and CXCR2^−/−^ mouse articular cartilage 8 weeks following DMM. Nuclei are counterstained with DAPI. Scale bar, 100 μm. (F) Densitometric quantification of ColX staining in articular cartilage following DMM (n=4) *p<0.05, **p<0.01, ***p<0.001.

To investigate whether ELR+ CXC chemokine signalling has a physiological function *in vivo*, we compared the outcome of experimental OA in CXCR2-deficient mice and wild-type controls following DMM surgery. CXCR2-deficient mice, which, at the time of surgery did not display histological features of OA and expressed collagens type II and type X at levels comparable to wild-type controls (see online supplementary figure S2), exhibited increased OA-like cartilage breakdown in DMM-operated joints in comparison to wild-type controls (figure 3A, B). In keeping with OA features, CXCR2-deficient mice also displayed lower expression of collagen type II within the articular cartilage (figure 3C, D), accompanied by increased expression of the chondrocyte hypertrophy marker collagen type X (figure 3E, F). These results confirm that CXCR2 signalling supports articular cartilage homeostasis *in vivo* in conditions of challenge and demonstrate that its disruption is associated with more severe OA.

### CXCR1/2 signalling is required for maintenance of articular chondrocyte phenotypic stability in a cell-autonomous fashion

**Figure 4:**
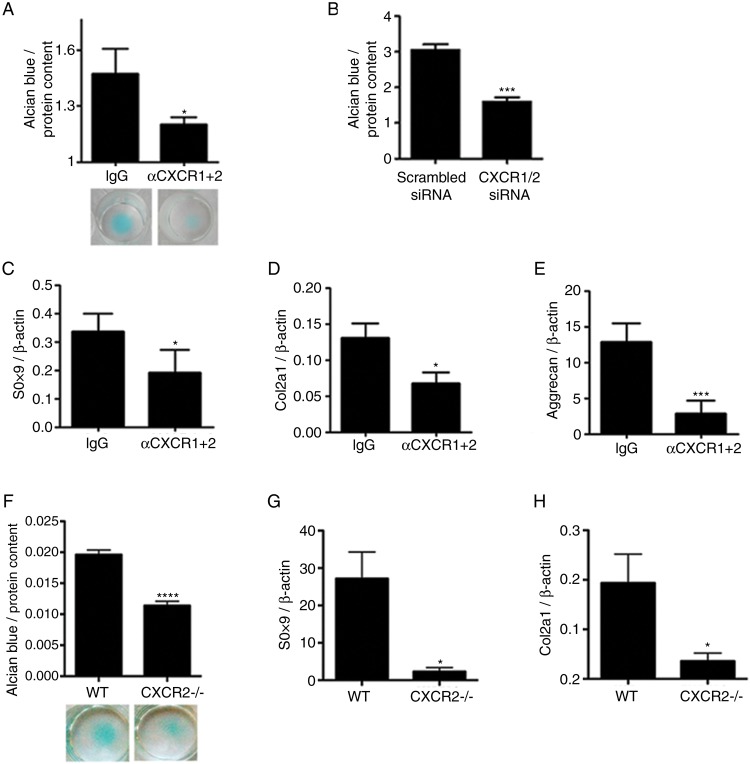
Disruption of CXCR1/2 signalling results in chondrocyte de-differentiation and reduced extracellular matrix production in a cell-autonomous fashion. (A) Alcian blue staining and spectrophotometric quantification for sulphated proteoglycan content of human articular chondrocyte micromass cultures 4 days following treatment with either CXCR1 and CXCR2 blocking antibodies or a non-specific IgG isotype control (n=4). (B) Spectrophotometric quantification of Alcian blue staining of JJ012 micromass cultures 4 days following CXCR1 and CXCR2 siRNA transfection compared with scrambled siRNA-treated control (n=4). (C–E) Real-time RT-PCR analysis of chondrocyte phenotype marker genes SOX9, COL2A1 and aggrecan expression in human primary chondrocytes 4 days following treatment with CXCR1 and CXCR2 blocking antibodies in comparison to non-specific IgG isotype control (n=4). (F) Alcian blue staining and spectrophotometric quantification of wild-type and CXCR2^−/−^ mouse costal chondrocytes cultured for 7 days in micromass (n=8). (G, H) Real-time RT-PCR analysis of SOX9 and COL2A1 mRNA expression of freshly isolated costal chondrocytes from wild-type and CXCR2^−/−^ mice (n=4). *p<0.05, ***p<0.001, ****p<0.0001.

OA is a disease of the whole joint and several mechanisms, both within and outside of cartilage, contribute to its pathogenesis. To investigate whether the homeostatic effects of CXCR2 signalling on chondrocytes are cell-autonomous, we investigated whether disruption of CXCR2 signalling *in vitro* in human chondrocytes resulted in phenotypic changes. Specific simultaneous inhibition of CXCR1 and CXCR2 using blocking antibodies in human primary chondrocytes (figure 4A), or by siRNA in the JJ012 human chondrosarcoma cell line (figure 4B), resulted in reduced ECM production in micromass cultures. Accordingly, treatment of human primary articular chondrocytes with anti-CXCR1 and CXCR2 blocking antibodies resulted in the downregulation of SOX9, COL2A1 and aggrecan mRNA in comparison to those treated with an isotype-matched IgG negative control (figure 4C–E).

In keeping with these data, freshly isolated costal chondrocytes from CXCR2-deficient mice displayed reduced sulfated proteoglycan accumulation compared with wild-type controls when cultured in micromass (figure 4F) and expressed significantly less SOX9 and COL2A1 mRNA (figure 4G, H). Therefore, cell-autonomous CXCR1/2 signalling in human, and CXCR2 signalling in mouse, are required for the maintenance of chondrocyte phenotypic stability.

### CXCR2-dependant modulation of the chondrocyte phenotype is AKT dependent

We then asked the question of what molecular mechanism links CXCR2 signalling to the maintenance of SOX9 expression and related phenotypic stability. It is known that AKT mediates both chemotactic CXCR2 signalling in neutrophils^31^ ^32^ and anabolic IGF-1 signalling in human chondrocytes.^33^ Therefore, we tested the hypothesis that CXCR2 signalling supports SOX9 expression via AKT activation.

**Figure 5:**
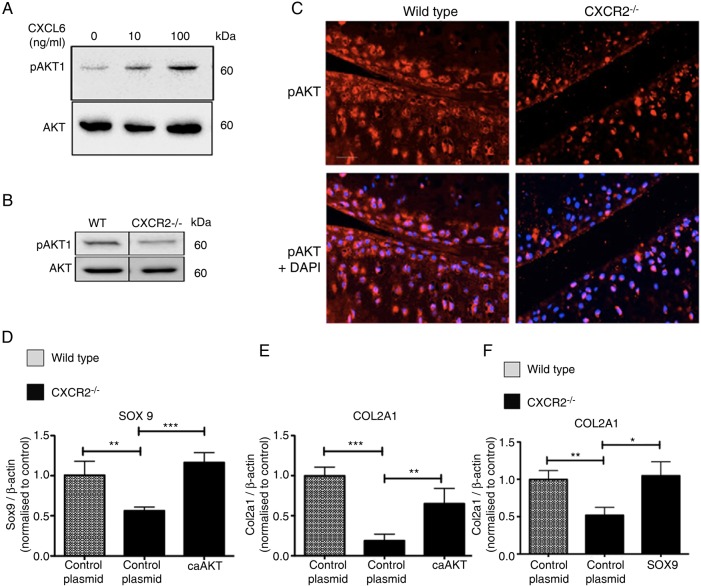
CXCR2 modulation of the articular chondrocyte phenotype is mediated by AKT. (A) Western blot of phospho-AKT (ser473) in wild-type mouse chondrocytes following 30 min incubation with recombinant mouse CXCL6. (B) Western blot comparison of phospho-AKT in freshly isolated chondrocytes from wild-type and CXCR2^−/−^ mice. (C) Immunofluorescence staining for pAKT in mouse articular cartilage of unchallenged wild-type and CXCR2^−/−^ mice, nuclei are stained with 4′,6-diamidino-2-phenylindole. Scale bar, 100 μm. (D, E) Real-time RT-PCR analysis of SOX9 and COL2A1 mRNA expression of wild type and CXCR2^−/−^ early passage mouse chondrocytes 24 h following transfection with either a control empty plasmid or constitutively active AKT (caAKT) expressing plasmid. (F) Real-time RT-PCR analysis of COL2A1 mRNA expression of wild-type and CXCR2^−/−^ mouse chondrocytes 24 h following transfection with either a control empty plasmid or a SOX9 expressing plasmid, *p<0.05, **p<0.01, ***p<0.001.

CXCL6 dose-dependently induced AKT phosphorylation in mouse primary chondrocytes (figure 5A). Less AKT phosphorylation was detected by western blotting in cell lysates obtained from CXCR2-deficient mouse costal chondrocytes in comparison to wild-type controls (figure 5B). *In vivo*, less phosphorylated AKT was detected in the articular cartilage of CXCR2-deficient mice compared with wild-type controls (figure 5C). In keeping with the decreased CXCL6 levels in OA, AKT phosphorylation was also decreased following DMM in wild-type mice (see online supplementary figure S3).

To assess whether CXCR2-induced AKT phosphorylation is required for chondrocyte differentiation or whether it is simply associated with it, we tested whether rescuing AKT activity also rescued the differentiation of CXCR2-deficient chondrocytes. Chondrocytes from wild-type and CXCR2-deficient mice were transfected with a constitutively active AKT (caAKT)-expression plasmid or empty plasmid as control. As expected, Sox9 mRNA expression was reduced in CXCR2-deficient chondrocytes compared with wild-type chondrocytes; however, SOX9 was rescued to levels comparable to those of wild-type cells following transfection with caAKT (figure 5D). The same pattern was observed for type II collagen (figure 5E). Transfection of CXCR2-deficient chondrocytes with a SOX9 expressing plasmid also resulted in the rescue of COL2A1 mRNA levels to levels comparable to that of wild-type cells (figure 5F).

**Figure 6:**
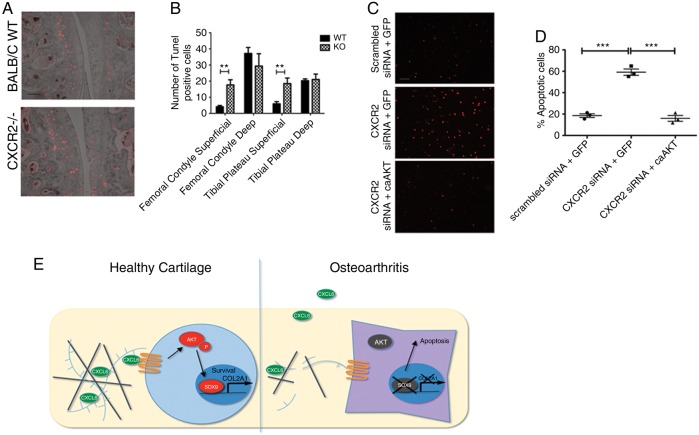
Disruption of CXCR2 signalling results in increased chondrocyte apoptosis in an AKT-dependent manner. (A) Terminal deoxynucleotidyl transferase dUTP nick end labelling (TUNEL) staining of wild-type and CXCR2^−/−^ articular cartilage 8 weeks following destabilisation of the medial meniscus surgery. Scale bar, 100 μm. (B) Quantification of TUNEL-positive chondrocytes in superficial and deep zones of articular cartilage of wild-type and CXCR2^−/−^ mice (n=5). (C) TUNEL staining of monolayer differentiated ATDC5 24 h following co-transfection with either scrambled control or CXCR2 siRNA along with either a control or caAKT expressing plasmid. Scale bar, 100 μm. (D) Quantification of TUNEL-positive ATDC5 cells following siRNA and plasmid transfection (n=3) **p<0.01, ***p<0.001. (E) In healthy articular cartilage, CXCL6 is expressed by chondrocytes and retained within the extracellular matrix (ECM) by HSPGs where it is available and required for signalling via CXCR1 and CXCR2 on nearby chondrocytes for the maintenance of their phenotypic stability. During osteoarthritis, mechanical and inflammatory injury leads to the breakdown of HSPGs within the ECM, leading to the release of CXCL6. This not only results in the release of CXCL6 from the articular cartilage, but disrupts the cell-autonomous ELR+ CXC chemokine signalling mechanism required for chondrocyte homeostasis.

Similarly to CXCR2 deficiency, SOX9 deficiency in adult chondrocytes does not result in spontaneous OA^34^ but makes chondrocytes more prone to apoptosis,^35^ ^36^ a process that has been demonstrated to drive cartilage loss during osteoarthritis.^37^ ^38^ Therefore, we tested whether CXCR2 disruption results in increased chondrocyte apoptosis in an AKT-dependent manner. First, at 8 weeks following DMM, CXCR2-deficient mice displayed significantly greater chondrocyte apoptosis within the superficial cartilage layers compared with wild-type mice (figure 6A, B). Although much lower than after DMM, the number of apoptotic cells in the superficial layer of control joints of CXCR2-deficient mice was significantly higher than in wild-type controls (see online supplementary figure S4). Second, siRNA-mediated knockdown of CXCR2 in the chondrogenic ATDC5 cells resulted in increased spontaneous apoptosis compared with scrambled siRNA control. Overexpression of caAKT, however, prevented the increase of apoptosis induced by the silencing of CXCR2 (figure 6C, D). Taken together, these data suggest that CXCR2 signalling protects chondrocytes from apoptosis in conditions of challenge by supporting AKT phosphorylation and SOX9 expression.

## Discussion

In this study, we discovered that CXCL6 is expressed by articular chondrocytes in physiological conditions and is retained locally in the cartilage matrix to contribute to the phenotypic stability and functional homeostasis by supporting SOX9 expression in an AKT-dependent manner. Disruption of CXCR2 signalling resulted, *in vivo*, in increased susceptibility to instability-induced OA, and *in vitro*, in loss of differentiation markers and ECM production.

Although no significant infiltration of inflammatory cells was detected 8 weeks following DMM in either CXCR2^−/−^ or wild-type mice (see online supplementary figure S5), this experimental set-up does not allow us to assess whether additional CXCR2 functions in cells other than chondrocytes contributed to the phenotype.

Our findings that CXCR1/2 signalling supports cartilage homeostasis are not at odds with the well-established pathogenic role of ELR+ CXC chemokines in arthritis.^7^ ^8^ ^39^^–^43 In physiological conditions, a tight regulation of their expression, together with their matrix binding through HSPGs, allows for the restriction of their signalling domain to the avascular chondrocyte pericellular matrix, away from the reach of inflammatory cells. In arthritis, ECM breakdown, together with the upregulation of multiple chemokines, including CXCL8,^42^ would result in excessive and ectopic activation of chemokine signalling in the joint with pathological consequences, while simultaneously depriving chondrocytes of homeostatic local chemokine signalling (figure 6E).

Interestingly, other chemokine families have been linked to physiological and even developmental roles outside of inflammation including several developmental processes^44^ ^45^ and the homeostasis of the haematopoietic system.^46^ This suggests that compartmentalisation of chemokine signalling in specific tissue contexts plays an important role in defining their function, and that in specific situations, the disruption of such compartmentalisation, rather than the expression of the chemokines themselves, may be pathogenic.

CXCR2-deficient mice did not develop spontaneous OA, but their phenotype was elicited after joint destabilisation. If CXCR1/2 signalling supports SOX9 expression, why did we not observe spontaneous osteoarthritis in CXCR2-deficient mice? In this respect it is interesting to notice that, although SOX9 is essential for embryonic chondrogenesis,^4^ its disruption in adulthood did not result in spontaneous OA;^34^ however, its absence from differentiated chondrocytes made them susceptible to apoptosis.^35^ ^36^ Therefore, SOX9 is strictly required for chondrogenesis, but, once chondrocytes are differentiated, it becomes only required in conditions of challenge. A second consideration is that, since SOX9 is upregulated following cartilage damage,^38^ ^47^ the baseline expression of SOX9 is sufficient to support cartilage homeostasis in physiological conditions, but is insufficient when, after cartilage damage, SOX9 upregulation is required.

It is interesting to note that the baseline phosphorylation of AKT was reduced upon inhibition of CXCR2 signalling. Since, in chondrocytes, AKT phosphorylation mediates IGF1 signalling, which is a potent homeostatic signal supporting chondrocyte differentiation and ECM production,^48^ this suggests a certain level of interaction between these two signalling pathways. The hierarchy of such interactions is yet to be determined.

The dual role of ELR+ CXC chemokines, homeostatic in healthy cartilage and pathogenic in arthritis, represents an important pharmacological challenge and yet an opportunity for the development of targeted strategies for cytokine blockade that preserve homeostatic mechanisms, while efficiently targeting the synovial and systemic compartments. The heterogeneity of mechanisms of cartilage damage in different subsets of patients is likely to require personalised therapeutic intervention, addressing individual disease mechanisms. We believe that further knowledge of three aspects of chemokine biology will be key to achieving this therapeutic goal: first, the mechanism by which signalling domains are defined and restricted; second, the role of ligand and receptor specificity in fine-tuning the regulation of chemokine signalling; and finally, the identification of suitable delivery systems to target intervention to specific tissue compartments.

## Supplementary Material

Web supplement

Web figures
