# Supplementary material for: A homeostatic function of CXCR2 signalling in articular cartilage
Source: Ann Rheum Dis. 2014 Aug 18;74(12):2207–15. doi: 10.1136/annrheumdis-2014-205546 (PMC4680121; doi:10.1136/annrheumdis-2014-205546)
Supplement: Web supplement [file annrheumdis-2014-205546-s1.pdf]

Supplementary Methods

Western blot analysis

Cells were lysed in RIPA buffer containing PhosSTOP protease inhibitor cocktail tablets (Roche) and Complete Mini EDTA–free phosphatase inhibitor cocktail tablets (Roche). 30µl of total cell lysates were run on a 10% Tris-glycine gel (Life Technologies) and transferred onto nitrocellulose membrane (GE Healthcare). Membranes were blocked for 3 hours in 5% BSA, 0.1% Tween20 PBS solution, then treated with rabbit anti-mouse pAKT (ser473) (Cell Signaling) 1:200 dilution, rabbit anti-mouse AKT (Cell Signaling) 1:500 dilution, or rabbit anti-mouse CXCL6 (Biorbyt) 1:200 dilution in blocking solution at 4°C overnight. Protein bands were detected using horseradish peroxidase conjugated secondary antibodies and chemiluminescent substrates (ECL Western Blotting Detection Reagents, Amersham).

Immunohistochemical analysis of mouse cartilage

Decalcified mouse knee joint sections were deparaffinized and dehydrated in xylene and 100% ethanol. Sections were blocked in 10% H_2O_2 in methanol, washed in PBS and digested in trypsin for 10 minutes. After further washing, sections were blocked in 10% horse serum in PBS for 10 minutes. The sections were incubated at 4°C overnight with Ly6B.2 primary antibody (AbD Serotec), 1:2000 dilution. After washing in PBS, sections were incubated for 30 minutes at room temperature with biotinylated goat anti-rat IgG (Vector Laboratories), 1:200 dilution, washed again and incubated for 30 minutes at room temperature with the Vectastain ABC peroxidase complex (Vector Laboratories) according to the manufacturer's instructions. After further washing, the staining was developed using DAB substrate (Vector Laboratories) according to the manufacturer's instructions and slides were mounted in DPX.

Additional immunofluorescence staining of human and mouse articular cartilage

Human cartilage paraffin sections were deparaffinised and stained as described in the Materials and Methods sections of the manuscript, using the following antibodies. CXCL8 staining was performed using mouse anti-human CXCL8 primary antibody (R&D), followed by Cy2 conjugated goat anti-mouse IgG secondary antibody (Jackson ImmunoResearch). Mouse CXCL1 staining was performed using rat anti-mouse CXCL1 primary antibody (R&D), followed by Alexa Fluor 488 goat anti-rat IgG secondary antibody (Life Technologies).

Safranin Orange staining

Human or mouse cartilage paraffin sections were deparaffinised as described in Materials and Methods. Sections were incubated with 0.2% Safranin Orange in acetate buffer for 13 minutes at room temperature, washed in distilled water, 100% ethanol and xylene and mounted in DPX.
