# Supplementary material for: A homeostatic function of CXCR2 signalling in articular cartilage
Source: Ann Rheum Dis. 2014 Aug 18;74(12):2207–15. doi: 10.1136/annrheumdis-2014-205546 (PMC4680121; doi:10.1136/annrheumdis-2014-205546)

Supplementary figures

Supplementary Figure 1. (A) Immunofluorescence staining comparison of CXCL8 (green) in healthy cartilage and preserved areas of OA cartilage. Nuclei are stained using propidium iodide. Scale bar, 100µm. (B) Safranin orange staining of healthy articular cartilage and preserved areas of OA articular cartilage. Scale bar, 100µm. (C) Immunofluorescence staining of mouse CXCL1 (green) in articular cartilage from sham and DMM operated mouse articular cartilage. Nuclei are stained using DAPI (blue). Scale bar, 100µm. (D) Real time RT-PCR for CXCL8 mRNA in early and late passage human articular chondrocytes (n = 3), **P < 0.01 by paired t-test.

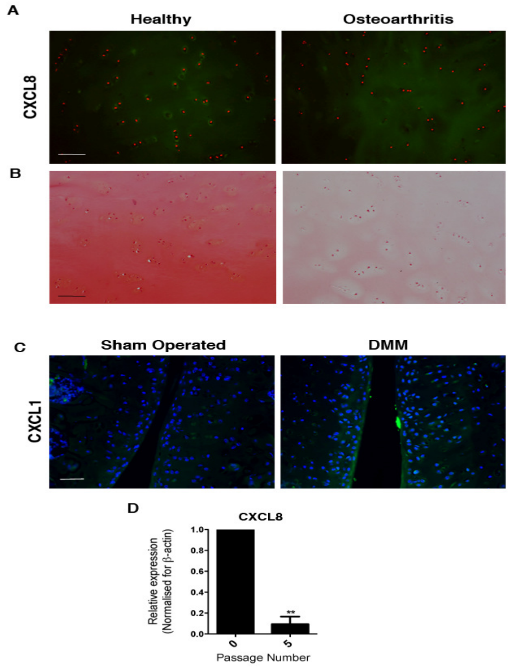

Supplementary Figure 2. (A) Histological comparison of 10 week old CXCR2- /- mouse articular cartilage to wild type controls stained using Safranin orange. Scale bar, 200µm. (B, C) Immunofluorescence staining and quantification of type II collagen and type X collagen (green) in unchallenged wild type and CXCR2-/- articular cartilage. Nuclei are stained using DAPI (blue). Scale bar, 100µm.

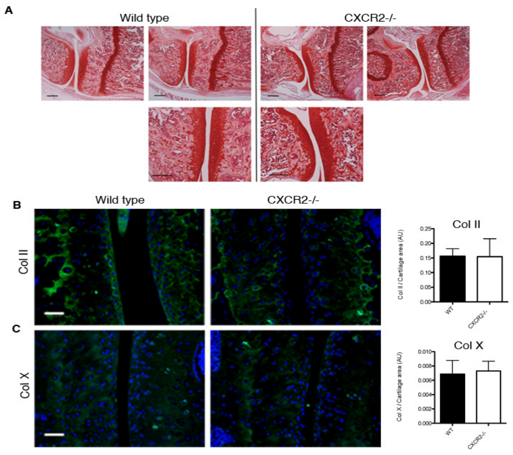

Supplementary Figure 3. Immunofluorescence staining of phospho-AKT (473) (red) in wild type sham operated control and DMM operated knees 8 weeks following surgery. Nuclei are stained using DAPI (blue). Scale bar, 100μm.

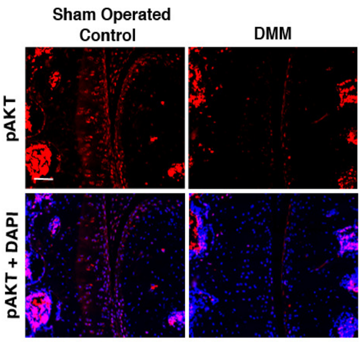

Supplementary Figure 4. (A) Quantification of TUNEL postive chondrocytes in superfical and deep zones of articular cartilage of unchallenged wild type and CXCR2-/- mice (n = 4). (B) Quantification of TUNEL positive in vitro cultured chondrocytes from wild type and CXCR2-/- mice (n = 4).

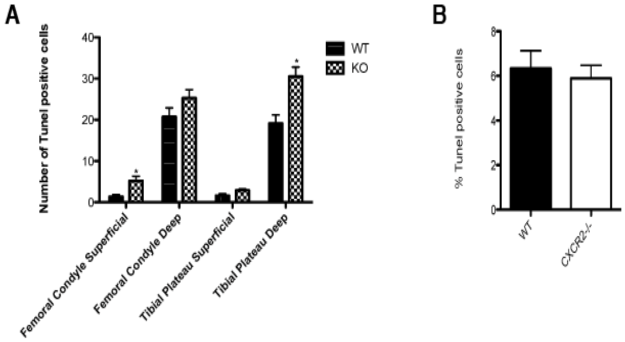

Supplementary Figure 5. (A) Immunohistochemical staining for Ly-6B.2 in hind paws of hTNFtg mutant mice. Scale bar, 100µm (B) Immunohistochemical staining for Ly-6B.2 in knee joints of wild type and CXCR2-/- mice 8 weeks following DMM surgery. Scale bar, 100µm.

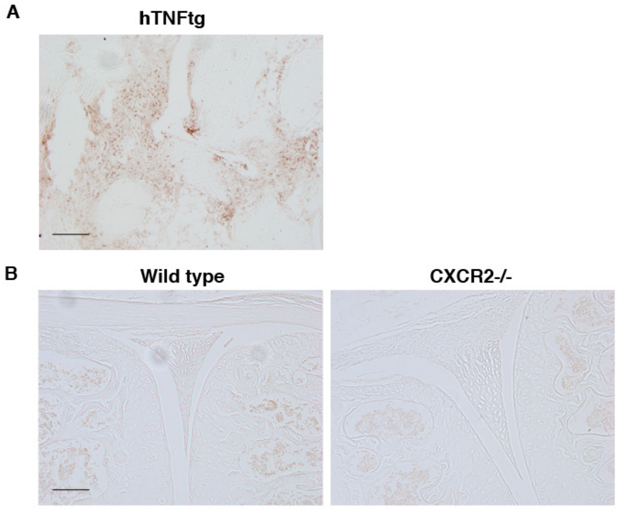

Supplement: Web figures [file annrheumdis-2014-205546-s2.pdf]
